# An Institutional Standardised Protocol for the Treatment of Acute Displaced Midshaft Clavicle Fractures (ADMCFs): Conservative or Surgical Management for Active Patients?

**DOI:** 10.3390/healthcare11131883

**Published:** 2023-06-29

**Authors:** Carlo Biz, Assunta Pozzuoli, Elisa Belluzzi, Davide Scucchiari, Nicola Luigi Bragazzi, Alessandro Rossin, Mariachiara Cerchiaro, Pietro Ruggieri

**Affiliations:** 1 Orthopedics and Orthopedic Oncology, Department of Surgery, Oncology and Gastroenterology DiSCOG, University-Hospital of Padova, Via Giustiniani 3, 35128 Padova, Italy; 2 Centre for Mechanics of Biological Materials, University of Padova, 35131 Padova, Italy; 3 Musculoskeletal Pathology and Oncology Laboratory, Department of Surgery, Oncology and Gastroenterology, University of Padova, Via Giustiniani 3, 35128 Padova, Italy; 4 Laboratory for Industrial and Applied Mathematics (LIAM), Department of Mathematics and Statistics, York University, Toronto, ON M3J 1P3, Canada

**Keywords:** clavicle, clavicle fracture, midshaft clavicle fracture, shortening, displacement, clavicle surgery, figure-of-eight bandage

## Abstract

Background and Objectives: The treatment of acute displaced midshaft clavicle fractures (ADMCFs) is still under debate. The aim of this study was to verify the effectiveness of our institutional protocol by comparing the clinical and radiographic outcomes of two groups of patients with ADMCFs treated operatively and non-operatively. Materials and Methods: active patients with a traumatic, isolated non-pathological ADMCF with at least 1-year clinical and radiographic follow up were included. Surgical treatment was performed in the cases where the residual displacement was higher than 140% after the application of a figure-of-eight bandage (F8-B). All other cases were treated conservatively with a F8-B. A total of 134 patients were enrolled and divided into two groups: surgical and conservative groups, with 59 and 75 patients, respectively. Radiological and clinical parameters were evaluated. Results: Good clinical (Constant-Murley Score, the Quick Disability of the Arm, Shoulder and Hand score, and VAS satisfaction) and radiographic outcomes (initial and residual shortening, initial and residual displacement) were obtained for ADMCFs in both groups. Multivariate analysis showed that patients treated conservatively had better clinical outcomes compared to surgically treated patients (*p* < 0.001). Return to sports was longer in those treated with surgery. Initial shortening was found to impact clinical outcomes as well as initial displacement. None of the patients showed signs of non-union in both groups. Conclusions: Very good mid-term clinical results can be obtained in adult patients with ADMCFs, conservatively or operatively managed, by applying our institutional treatment protocol based on objective radiographic parameters evaluated in the ER.

## 1. Introduction

Clavicle fractures are the most common bone injuries after distal radius fractures (17% of all fractures) [1,2]. They account for 2.6–4% of all fractures and represent 34–35% of shoulder girdle injuries [1,2]. Approximately 82% of them, often displaced, affect the clavicle midshaft and generally occur in young and middle-aged active people [1]. Traffic accidents, accidental falls and sports activities are the most common cause of clavicle fractures as a consequence of a fall on an outstretched hand or on the shoulder, or of a direct hit to the shoulder [3]. Sports-related clavicle fractures represent approximately 30% of all clavicle fractures and are increasing due to the growing number of persons involved in sports and recreational activities [4,5]. Clavicle fractures account for up to 10% of all sport-related fractures, and approximately 30% of all clavicle fractures happen during sport activities [4]. Furthermore, athletes affected by these fractures have not only the third longest return time to sports compared to patients with distal radius fractures and tibial diaphysis fractures, but about 20% of them fail to return to sports activities [6].

The current care in acute clavicle fractures is either operative or non-operative treatment. Non-operative management of non-displaced acute midshaft clavicular fractures (MCFs) using bandage immobilisation or sling is satisfactory, while the treatment of displaced fractures is still controversial [7,8,9,10]. At present, clavicular plating remains the gold standard for operative treatment of displaced ADMCFs as most of the devices previously proposed for internal fixation are less used, such as intramedullary titanium elastic nails, Rockwood pins, Kirschner wire, rush nail and Küntscher nails [11]. The main reason for the unpopularity of these other devices is removal of the implant after fracture union. Several authors have reported on the functional outcomes of both operative and non-operative management of ADMCFs [12,13,14,15,16]. However, the literature has failed to conclusively demonstrate the best indication for these injuries.

In 2020, a systematic review and network meta-analysis of randomised controlled trials regarding ADMCF management concluded that surgery for displaced fractures can increase bone union but does not guarantee better functional outcomes than conservative treatment. On the other hand, most of these injuries can be treated non-operatively with little absolute risk of non-union as reported by Axelrod et al. [9]. Nevertheless, non-union is more difficult to manage than an acute dislocated fracture [9].

Recent studies, however, have demonstrated that non-union and malunion rates with non-operative treatment are greater than the rates believed in the past, especially for displaced fractures, thus suggesting the need for a different treatment [17].

For athletes, the currently prevailing opinion is that conservative management of acute displaced clavicle fractures (ADMCFs) results in increased time to return to sports, often without return to pre-injury levels. This is due to worse shoulder function caused by malunion and clavicle shortening with thoracoscapular dyskinesia, as well as increased re-injury rates [4]. Accordingly, surgical treatment has increased in recent years because it has been shown that there is a faster return to sports, better return to pre-injury sports ability and improved shoulder function, despite some controversies regarding the relatively high complication rate (≥23%) [18], such as infection, non-union and implant failure [19].

Among the main advantages of surgery reported, there is a low rate of non-union [20,21] and immediate fracture stability, which provides early post-operative mobilisation [22] and better functional outcomes at 6-month follow up [4]. However, current studies do not show differences in functional outcomes at one year follow up between conservative treatment and plate fixation of ADMCFs [4,13]. Van der Ven, Denise et al. reported similar functional results after 24 weeks as well as after 5 years of follow up [23]. Furthermore, surgical fixation is associated with complications in up to 29% of patients, including wound infections, neurological symptoms, frozen shoulder and implant-related problems [20].

Nevertheless, in the present era of shared decision making, the potential benefits of surgery should be carefully analysed in relation to complications and costs [4,5,8]. To date, there are few absolute indications for early surgical fixation: open fractures, neurological deficiencies, compromised skin conditions, vascular injury, ipsilateral serial rib fractures, floating shoulder, widely displaced fragments and comminuted fractures. This is because most ADMCFs are managed successfully by conservative means with good to excellent clinical outcomes [24]. For these reasons, surgery becomes the treatment of choice in case of failure of conservative treatment [25,26].

Recently, residual displacement (RD), measured after figure-of-eight bandage (F8-B) application, was identified as a predictive factor of delayed union and non-union for patients with ADMCFs non-operatively treated (RD of 104% for delayed union and 140% for non-union) [27]. Based on these findings, a protocol for the treatment of ADMCFs was developed at our institution whose strict application showed that in patients with a RD of less than 104%, good to excellent clinical outcomes could be achieved without non-union when treated conservatively [28].

The aim of this study was to verify the effectiveness of our institutional protocol by comparing the clinical and radiographic outcomes of two groups of patients with ADMCFs treated operatively and non-operatively. We hypothesised that our institutional protocol could be a valid tool to help orthopaedic surgeons choose the most appropriate option, conservative or surgical, for the treatment of ADMCFs from early diagnosis in the emergency room (ER).

## 2. Materials and Methods

### 2.1. Study Design

This study was designed as an observational single-centre retrospective case series, including patients affected by an ADMCF between December 2016 and December 2018. Patients were enrolled in the study after receiving a thorough explanation of the risks and benefits of inclusion and providing their written informed consent. The study was approved by the Institutional Ethics Committee (CESC code 319n/AO/22, 15 December 2022) and was performed in accordance with the ethical standards of the 1964 Declaration of Helsinki as revised in 2013 and those of Good Clinical Practice. Inclusion criteria were as follows: active patients with a traumatic, isolated non-pathological ADMCF, between 18 and 80 years old, having followed our institutional protocol for the treatment of their ADMCF, including the rehabilitation program, with at least 1-year clinical and radiographic follow up. Exclusion criteria were patients with an open, stable, undisplaced MCF who had previous injuries of the clavicle or delayed surgery, patients with a bilateral clavicle fracture, patients with significant comorbidities (i.e., rheumatological, oncological, neurological or cognitive types, and systemic infections), patients who refused suggested treatment, polytraumatic patients (having fractures at multiple sites).

Patients were divided into two groups according to their treatment indications: conservative group, patients treated conservatively and surgical group, patients who underwent surgery within 3 weeks from injury.

### 2.2. Patients

At our level-1 healthcare trauma centre, a standardised institutional treatment protocol for patients with ADMCFs was adopted based on our previous experience and applied methodologically as has already been described in the literature [27,28,29].

**Figure 1:**
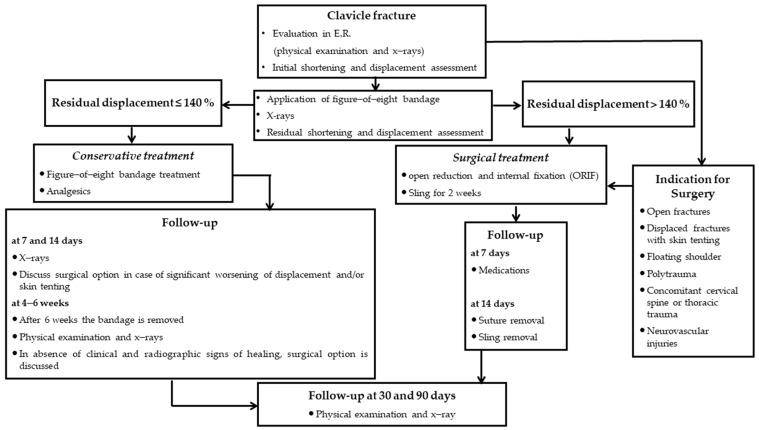
Algorithm of ADMCFs treatment.

Briefly, a trauma surgeon of our unit first evaluated patients in the ER with a complete clinical examination to evaluate functional impairment of the shoulder, associated injuries of the brachial plexus or the subclavian vessels [30] and chest complications, such as the pneumothorax or hemothorax [31,32,33,34]. The diagnosis was then confirmed with plain X-rays (standard anteroposterior and 20° cephalic tilt views). Then, an F8-B was applied to all patients of our cohort who then underwent immediate radiographic control to check that fragment alignment was achieved. In case of severe RD > 140% after F8-B application, or when reduction was impaired by mechanical factors (soft tissue interposition, comminution or vertical fragments), surgery was suggested (Figure 1). This is because RD was identified as a predictive factor of delayed union and non-union for patients with ADMCFs non-operatively treated (RD of 104% for delayed union and 140% for non-union) in our previous study [27].

#### 2.2.1. Conservative Group

Patients included in the conservative group and treated with F8-B were thoroughly instructed on the bandage’s use and its correct positioning to avoid axillary decubitus ulcers and compression of the neurovascular bundle.

In the first phase, these patients were instructed to avoid active movements of the shoulder. Only passive range of motion (ROM) below 90° forward flexion and slight movements of the hand and the elbow (without load) were permitted [35].

Patients treated conservatively then underwent clinical and radiographic assessments at 7 days and 14 days after trauma to evaluate F8-B tolerability and position. In cases of significant worsening of displacement and/or skin tenting, surgery treatment was discussed with the patient. The F8-B was maintained from 4 to 6 weeks depending on fracture healing. During this period, the patients were allowed to perform only passive shoulder movements, below 90° forward flexion and slight movements of the elbow and wrist (without load).

When the F8-B was removed after radiographic control, patients were trained to perform Codman exercises and strengthening to gradually increase active shoulder movements and to achieve full ROM in 3 to 4 weeks with the assistance of the same rehabilitation team of our institution [36]. After clinical and radiological fracture healing, weightlifting, contact sports and heavy physical activity were allowed.

#### 2.2.2. Surgical Group

Surgical treatment was suggested and performed in those cases where the RD was higher than 140% after the application of an F8-B. All other cases were treated conservatively with an F8-B.

In all operations, an interscalene brachial plexus block and a laryngeal mask airway anesthesia were performed. Prophylactic cefazolin (2 g) was administered and continued 24 h after surgery. All operations were performed by one of the trauma surgeons of our unit, including the senior authors.

The patients were treated in a standardised manner with open reduction and internal fixation (ORIF) as follows: a skin incision of 10–15 cm centred on the fracture site and a careful dissection of subcutaneous tissue and clavipectoral fascia was performed, completely exposing the fracture. Using a pointed clamp, the fragments were aligned and temporarily immobilised. Then, a 3.5 mm antero-superior clavicle LCP (Locking Compression Plate, de Puy-Synthes, Raynham, MA, USA) was fixed with screws on the anterosuperior aspect of the clavicle, taking care not to damage ligament and muscular insertions. Anatomical implants reducing soft tissue intra-operative damage provide a greater chance of restoring anatomical alignment, preserving muscle length and preloading, and decreasing the rate of non-union and surgical wound problems [19]. Intraoperative fluoroscopic evaluation was performed during and at the end of surgery to confirm reduction and correct implant fixation.

Patients were instructed to maintain the operated arm in a sling for 2 weeks. During this period, patients were allowed to perform only gentle passive shoulder, elbow and wrist movements to prevent joint contractures and oedema. After suture removal at the 14th day, they were trained to perform Codman exercises [20] and strengthening, while gradually active assisted shoulder movements below 90° forward flexion with a physiotherapist were allowed after X-ray control at one month from surgery. Then, patients were encouraged to reach full ROM of the arm during intensive physiotherapy sessions performed by the same rehabilitation hospital team of our institution but avoiding heavy weightlifting and resistance training exercises until the third month. Full return to activities was allowed once radiographic and clinical fracture healing was achieved.

### 2.3. Patient Assessment

External and independent investigators (D.S. and A.R.), not involved in the patients’ treatment, performed data collection retrospectively reviewing hospital records. Baseline characteristics of all patients were recorded including the following socio-demographic and clinical data: age, gender, body mass index (BMI), smoking habits, mechanism of trauma, affected/dominant side involved.

Radiographic fracture features were evaluated on standard X-rays performed at patient admission in the ER as follows: type of fracture type according to Classification of the Association for Osteosynthesis/Orthopedic Trauma Association (AO/OTA) [37]; initial shortening (IS) and residual shortening (RS), measured before and after the F8-B application, respectively, based on the overlap of proximal and distal fragments and expressed as a percentage of the same clavicle length on the antero-posterior view; initial displacement (ID) and residual displacement (RD), measured before and after the F8-B. Fragment displacement was defined as the amount of vertical translation and measured as a percentage of the clavicle width at the fracture site on a 20° cephalic tilt view of the clavicle [27,28]. Intra-reader and inter-reader reliability were found to be good (>0.80) for all measurements.

Clinical follow up was performed 7 and 14 days after treatment and afterwards at 1, 3, 6 and 12 months after trauma, and at last follow up. At the last follow up, functional outcomes were evaluated by the Constant–Murley Score (CS) [38] and the Quick Disability of the Arm, Shoulder and Hand score (qDASH), including the qDASH work and sports modules [39]. CS is composed of four items: pain, activities of daily living (ADL), ROM and strength. CS ranges from 0 (worst function) to 100 (optimum function). The qDASH score ranges from 0 (the best function) to 100 (the most disability and dysfunction).

Time of return to work and return to sports or recreational activities were evaluated, while patient satisfaction of their shoulder function was assessed by the visual analogue scale (VAS) (range 0–10).

Any complications were also recorded.

### 2.4. Statistical Analysis

Sample-size determination and power analysis were conducted by using G*Power software (version 3.1.9.7; Heinrich-Heine Universität Düsseldorf, Düsseldorf, Germany). A medium-large effect size (f = 0.30) between the two groups (conservative versus surgical treatment) was assumed, with an adjusted alpha error probability of 0.05 and a power of 0.80. The total needed sample size was found to be 115 patients.

An initial descriptive statistical analysis was conducted by an independent statistician of another institution. Continuous data were synthesised as means, standard deviations and medians (with their 25th and 75th percentiles or interquartile range, IQR) when appropriate. Categorical (nominal or ordinal) data were expressed as absolute and relative counts (percentages). Before proceeding with statistical processing and analysis, data were visually inspected for potential outliers. Data univariate normality distribution was checked by carrying out the Shapiro–Wilk test, whereas homogeneity of covariance matrices and multivariate normality were verified by means of Cox’s M test, residual analysis, the Shapiro–Wilk test and visual inspection of the Q-Q plot. The Shapiro–Wilk test for univariate normality was chosen, taking into account the sample size employed in the present investigation.

Data were computed for the entire study population and then stratified according to the type of treatment (conservative treatment group versus early surgery group). Univariate equality (or homogeneity) of variance between the two groups was verified by means of Levene’s test. Both univariate analysis (Student’s t-test for independent samples, Mann–Whitney rank-sum test for independent samples, chi-squared tests) and multivariate analyses of covariance (MANCOVA) were performed. For MANCOVA, a comprehensive set of metrics (Pillai’s Trace, Wilks’ Lambda, Hotelling’s Trace and Roy’s Largest Root) was computed. To ensure robustness against MANCOVA underlying assumptions, we relied on Pillai’s Trace, following Olson [40].

To analyse the impact of treatment type (early surgery versus conservative management) more interpretable and easily understandable, each outcome variable was dichotomized at its median. Binomial logistic regression models were then run, and the final effect-size was expressed as odds-ratio (OR), along with its 95% confidence interval (CI).

All statistical analyses were conducted using the commercial “Statistical Package for Social Sciences” (SPSS) software (SPSS for Windows, version 28, IBM Corporation, Armonk, NY, USA). A cut off of 0.05 for *p*-values was chosen to indicate statistically significant findings.

## 3. Results

### 3.1. Patients

**Figure 2:**
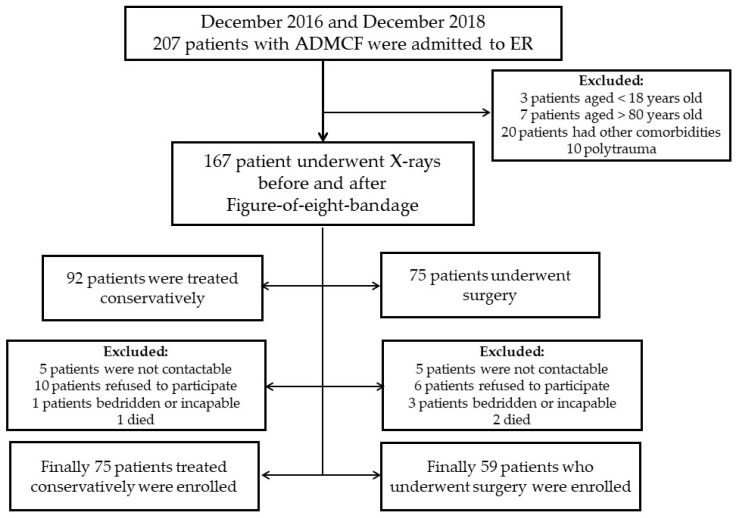
Study flowchart.

A total of 134 patients were enrolled in the present study (Figure 2).

Patients’ socio-demographic, clinical and radiological characteristics are reported in Table 1.

Mean age was 44.20 ± 14.80 years, most of the patients were males (85.1%) and the average body mass index (BMI) was 24.63 ± 2.55. Most of them were inactive smokers (55.2%). The most common type of trauma was bike fall (35.8%), followed by motorcycle trauma (28.4%), sports injury (20.1%) and simple fall (15.7%). In 46.3% of the cases, the trauma affected the dominant side. The most commonly reported fractures were of types B2 and B3 (21.6% and 32.8%, respectively).

The average IS and RS were 6.28 ± 5.14% and 4.62 ± 4.60%. Average ID and RD were 121.43 ± 42.48% and 102.63 ± 35.85%, respectively.

Regarding the clinical outcomes, an average total CS of 95.34 ± 6.24 and total qDASH of 4.87 ± 7.15 were reported (Table 1). According to Subramanyam et al. [41], CS was very good (86–100 points) in 123 patients (91.8%) and good in 11 (8.2%); no patients with fair (56–70 points) and poor (<56 points) scores were present. According to Angst et al. [42], qDASH corresponded to “no problem” in 124 patients (92.54%), “problem, but working” in 9 patients (6.71%) and “unable to work” in 1 patient of the surgical group (0.75%).

Mean times of return to work or sports were 2.61 ± 0.97 months and 4.45 ± 1.85 months, respectively. Mean patient satisfaction was 7.59 ± 1.08. The mean follow-up time was 29.6 ± 8.1 months.

Stratifying according to the type of trauma in the univariate analysis, patients with sports traumas were younger (31.00 ± 10.41 versus 47.53 ± 13.88 years, *p* < 0.0001) and less likely to be active smokers (*p* = 0.0281). No other differences could be detected in terms of socio-demographic and clinical as well as radiological and treatment variables. In terms of outcomes, patients with sports traumas were more likely to display greater total CS (97.26 ± 4.49 versus 94.86 ± 6.54, *p* = 0.0394), with strength (*p* = 0.0613) subscale exhibiting greater scores in a statistically borderline significant fashion (Supplementary Table S1).

### 3.2. Conservative Versus Surgical Treatment

**Figure 3:**
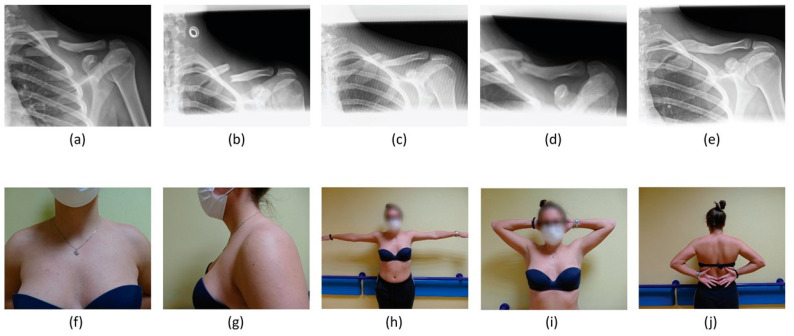
A 24-year-old female patient treated conservatively for an AO A3 left ADMCF. Clavicle radiographic images (**a**) at the time of patient presentation in ER, (**b**) at immediate post-reduction radiographic control using a F8-B, (**c**) at 30-day follow up, (**d**) at 6-month follow up and (**e**) at last follow up of 31 months. Last follow-up clinical images showing (**f**,**g**) no mass in the supraclavicular fossa and good range of movement in abduction (**h**), extra-rotation (**i**) and intra-rotation (**j**).

**Figure 4:**
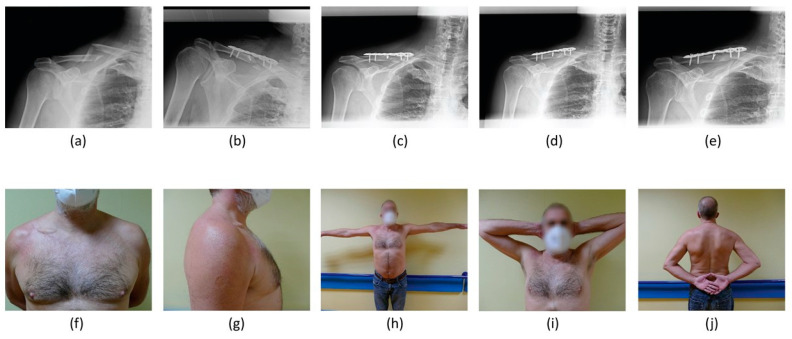
A 59-year-old male patient surgically treated for an AO B2 right ADMCF. Clavicle radiographic images at (**a**) the time of patient presentation in ER, (**b**) immediate post-operative radiographic control, (**c**) 30-day follow up, (**d**) 6-month follow up and (**e**) last follow up of 22 months. Last follow up clinical images showing (**f**,**g**) a mass of modest size on the right clavicular profile and patient’s satisfactory range of movement in abduction (**h**), extra-rotation (**i**) and intra-rotation (**j**).

Concerning management, 75 (56.0%) were treated conservatively, while 59 (44.0%) patients underwent surgery (Figure 3 and Figure 4).

In the univariate analysis, no differences were reported regarding age, gender, smoking habits, type of trauma and dominant side. BMI was higher in patients undergoing surgery (*p* = 0.0027). In terms of radiological variables, differences could be detected for type of fracture (*p* = 0.0251), IS (*p* = 0.0316) and RS (*p* = 0.0035), ID (*p* = 0.0087) and RD (*p* < 0.0001). In terms of outcomes, significant differences could be found for all parameters under study except for total and sports qDASH scores (*p* = 0.1466 and *p* = 0.0846, respectively). Surgically treated patients returned to work and sports later than conservatively treated patients (*p* = 0.0420 and *p* = 0.0011, respectively). VAS satisfaction did not differ between the two groups (*p* = 0.5798). Further details are shown in Table 2.

Regarding complications, eight patients treated conservatively reported re-fracture of the clavicle. These fractures occurred after traumas when clinical and radiographic healing was complete. Therefore, they should be considered as new injuries. Re-fractures are generally surgically treated at our institution; however, five patients refused surgery, while three underwent osteosynthesis by plate.

**Figure 5:**
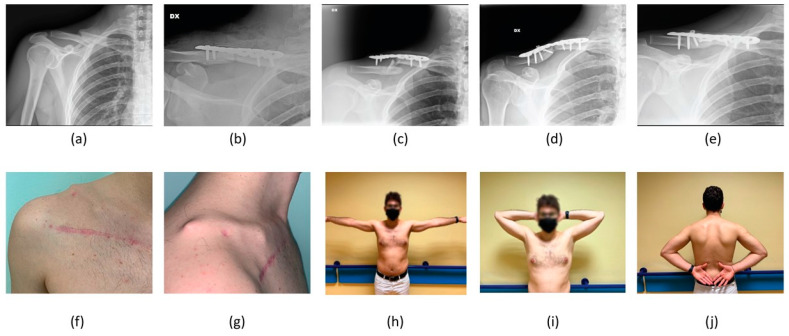
A 27-year-old male patient underwent surgical treatment twice for an AO A2 right ADMCF. Clavicle radiographic image at (**a**) the first time of patient presentation in ER, (**b**) immediate post-operative period, (**c**) after a second presentation in ER showing lateral screw loosening and dorsal displacement of the plate 4 months after the operation, (**d**) immediately after the second osteosynthesis and (**e**) at last follow up of 30 months. Clinical images of the patient presentation in ER for the implant failure showing (**f**,**g**) considerable swelling in the supraclavicular region. Last follow-up clinical images showing good range of patient movement in abduction (**h**), extra-rotation (**i**) and intra-rotation (**j**) with the plate still on site.

One patient in the surgical group presented implant failure for lateral screw loosening and consequent dorsal displacement of the plate, which was treated successfully by reoperation (Figure 5). Further, 5 patients reported infections that were successfully treated by antibiotics and implant removal. Plate removal was performed on 20 patients due to intolerance. Finally, no vascular or nerve lesions and non-union were reported.

### 3.3. Multivariate Analysis

Overall, in the multivariate analysis, the type of treatment (*p* = 0.012) resulted statistically significant, impacting the outcomes, while the type of fracture (*p* = 0.056) resulted statistically borderline. The IS (*p* = 0.088) and the ID (*p* = 0.147) failed to achieve the statistical significance threshold (Table 3 and Supplementary Table S2).

The type of treatment impacted most of the variables under study except for qDASH, including total (*p* = 0.183), work (*p* = 0.120) and sports (*p* = 0.364), as well as return to work (*p* = 0.197) and VAS satisfaction (*p* = 0.468) (Table 4 and Supplementary Table S3).

The type of treatment affected all CS domains (total CS *p* < 0.001, pain *p* < 0.001, ADL, *p* = 0.003, ROM *p* = 0.018, strength *p* < 0.001) with higher values in the conservative group. Return to sports was longer in those treated with surgery (*p* = 0.006). Similar trends could be reported when dichotomizing the outcome variables. The effect sizes of the impact of the type of treatment (early surgery versus conservative management) on each outcome variable are shown in Table 5. Those who underwent early surgery had a higher likelihood of reporting lower values in all CS domains (pain, OR 0.02 [95% CI 0.00 to 0.13]; ADL, OR 0.01 [95% CI 0.00 to 0.13]; ROM, OR 0.11 [95% CI 0.02 to 0.52]; strength, OR 0.00 [95% CI 0.00 to 0.03]; total CS, OR 0.04 [95% CI 0.01 to 0.21]). They also tended to display higher qDASH values (qDASH, OR 5.47 [95% CI 1.48 to 20.18]; qDASH work, OR 15.05 [95% CI 3.44 to 65.93]; qDASH sport, OR 22.18 [95% CI 4.72 to 104.13]) and a longer return to sports (OR 9.35 [95% CI 2.18 to 40.03]).

The type of fracture impacted ROM (*p* = 0.002), strength (*p* = 0.006) and total CS (*p* = 0.019). VAS satisfaction (*p* = 0.059) was impacted in a statistically borderline fashion. The IS influenced all of the CS domain values, including pain (*p* = 0.025), ADL (*p* = 0.034) and total CS (*p* = 0.008), with the exception of ROM (*p* = 0.111) and strength (*p* = 0.215), with an inversely proportional relationship (i.e., higher IS values corresponded to lower CS domain values). This impacted qDASH as well: total (*p* < 0.001), work (*p* = 0.010), sports (*p* = 0.042) and return to work (*p* = 0.023) in a directly proportional fashion, and VAS satisfaction (*p* = 0.012) in an inversely proportional fashion. The ID influenced strength (*p* = 0.016) and total CS (*p* = 0.042), both in an inversely proportional fashion (i.e., higher ID values corresponded to lower strength and total CS values).

## 4. Discussion

The most important finding of the present study is that good clinical and radiographic outcomes were obtained by applying our standardised protocol of treatment for ADMCFs in both groups, without non-union, the most frequent complication of these injuries. The treatment of ADMCFs remains controversial. Arguments in favor of conservative treatment are a lower rate of complications and comparable clinical outcomes at follow up [21,24,43,44]. Abdulaziz et al. pointed out that surgically treated patients had elective plate removal, while non-surgically treated patients had more surgical fixations for non-unions [17].

Nevertheless, both conservative and operative management have been recommended for ADMCFs, but no algorithm to choose between the two has been proposed so far, making it often difficult for orthopaedic surgeons to determine the best treatment for each subject.

Hence, the aim of this study was to verify the effectiveness of our institutional protocol, previously described [27,28], in the decision-making treatment for ADMCFs by comparing the clinical and radiographic outcomes of two groups of patients after conservative and surgical treatment.

Both the conservative group and the surgically treated group displayed very good CS (higher than 90) and qDASH values. A slightly better CS and relative subscales were found for the conservative group compared to the surgical one (96.77 vs. 93.53). However, these values are still lower than those generally defined as the minimal difference for the clinical relevance [45,46]. Furthermore, age, gender, smoking habits, type of trauma and dominant side involved were comparable between the two groups, while there was a difference concerning type of fracture (AO/OTA), IS, RS, ID and RD. All of these clinical and radiographic parameters were higher in the surgical group.

Importantly, the type of treatment was confirmed to significantly impact the clinical outcomes (total CS and subscales), including in multivariate analysis, in favor of conservative treatment. Nevertheless, all patients of both groups were satisfied, and this result could be related to the good functional outcomes achieved.

Van der Ven, Denise et al. reported that operative plate fixation of ADMCFs has an early effect on decreasing pain and improving function [23]. This is demonstrated by the significantly improved DASH and CS in the operative group observed at 6 weeks after injury compared to conservative treatment, but this difference was not retained at 24 weeks and 5-year follow up. Robinson et al. and the Canadian study group found an early significant benefit from plate fixation compared to non-operative treatment, supporting the use of surgery for ADMCFs in active adults [21,36]. However, these authors did not assess their results at longer follow up. In line with our findings, a recent meta-analysis showed no difference in functional outcome at 24 weeks of follow up and no clinically important difference in functional outcome at 5 years follow up for both treatment groups. These authors concluded, in agreement with two meta-analyses, that most patients experienced similar functional outcomes regardless of whether they were treated operatively or conservatively [9,10]. In a recent systematic review, Martin (2021) et al. found that compression plating resulted in significantly less disability early after surgery compared to nonoperative management [47]. This better functional outcome is not retained at one year of follow up, in accordance with our data at almost 30-month mean follow up.

To our knowledge, this is the first study that assesses several radiographic features (i.e., IS, RS, ID and RD) of ADMCFs at the time of trauma in both groups, conservatively and operatively managed. It should be noted that one of the major challenges is not only the different methods used to evaluate the radiological parameters but also the different timing of measurement (i.e., at the trauma or after union). Therefore, it is necessary to standardise both the methods and the timing of measurements to clarify the role of shortening and displacement in clavicle fractures.

Since both IS and RS play an increasingly important role in deciding on surgical intervention of ADMCFs, it is important to have a reliable and accurate method of measuring them [48]. However, the results of a recent systematic review demonstrate that the literature on this topic yielded only fair- and poor-quality studies. In contrast to current standard practice in which AP and 15° caudo-cranial views are made, we have been supporting the use of a 15–30° cranio-caudal AP (about 20°) view as being the most accurate in measuring the shortening of MCFs [49]. Supine positioning of the patient may underestimate the actual shortening and displacement, which in turn can negatively influence the decision to carry out surgical reduction and fixation of the ADMCFs. Standard X-rays of the injured clavicle are sufficient to evaluate fracture pattern, without needing contralateral clavicle views or making use of more refined, but expensive, radiological exams, such as CT scans, as proposed by other authors [48,49].

In our previous study, we showed that IS and RS correlated with the clinical outcomes [28]. The IS has not been shown to be a predictive risk factor either of delayed union or of non-union in our previous cohort of patients, supported by the data published by Jørgensen et al. [22]. Moreover, RS was found to be a predictor of functional outcomes [28].

In this study, all of the radiological features measured resulted significantly lower in the conservative group compared to the surgical one. Specifically, IS was found to have an impact on the clinical outcomes. Higher IS determined worse clinical outcomes (lower values of total CS, pain, ADL and higher values of qDASH), lower VAS satisfaction and a delay in the return to work, with multivariate analysis. Our data are supported by other studies that found an association between shortening and worse clinical outcomes or patient dissatisfaction [50,51]. In contrast, Woltz et al. published a systematic review evaluating the impact of shortening on shoulder function in patients treated conservatively. The conclusion was that the included studies did not allow adequate definition of the influence of shortening on shoulder function. The main issues of this review were the heterogenicity of the included studies. In particular, shortening was evaluated in different ways and at different time points [52]. A systematic review did not find any association among IS and functional outcomes [53]. Rasmussen et al. and Nordqvist et al. found no correlation between shortening (evaluated during the follow up) and clinical outcomes of patients treated conservatively [54,55]. 

Regarding clavicle displacement, no studies have focused on RD. Studies report only the evaluation of the presence of displacement in the ER without quantifying it. In our previous study, we observed that only RD can predict fracture healing [27], but we did not show a correlation with the clinical outcome of these patients [28]. Specifically, an RD ranging between 104 and 140 was a predictor of delayed union while an RD > 140 predicted non-union [27]. Here, multivariate analysis also highlighted an impact of ID on the clinical outcomes, independent of the type of treatment. Particularly, a higher ID is associated with a worse clinical outcome in terms of total CS and strength. In our study, the type of fracture has an impact on total CS, ROM and strength, independent of the type of treatment.

In our cohort, patients treated conservatively returned earlier to work and to sports compared to patients treated operatively. Our data are in contrast with other studies suggesting that surgical treatment allows earlier return to sports, in particular for athletes [4,56,57]. This result is probably related to the higher RD in the patients treated surgically based on our protocol. Hoogervorst et al. reported that patients treated conservatively or surgically, with non- or minimally displaced MCFs, had the same rate of return to sports [43].

No cases of non-union were recorded in our study, suggesting that the protocol applied in our institution seems to be effective in preventing this complication. Considering that in the literature non-union rates are reported to be between 5% and 20% after nonoperative treatment [9,10,53,58,59,60,61], we consider our results good.

The main limitation of this study is its retrospective design, which is susceptible to biases. Another shortcoming of the study is the relatively small number of patients enrolled in each group, which is still higher compared to other published studies [35,62,63,64]. However, all subjects enrolled followed the indications provided by the treatment protocol after ADMCF diagnosis and completed the rehabilitation program suggested by our multidisciplinary team.

Finally, to the best of our knowledge, this is the first study that assesses and compares functional outcomes evaluated at more than 2 years mean follow up and standardised radiographic features measured at the ER and just after treatment.

## 5. Conclusions

In accordance with our hypothesis, the present study shows that good to very good mid-term clinical results can be obtained in active adult patients with ADMCFs, conservatively or operatively managed, by applying our institutional treatment protocol, which is based on objective radiographic parameters previously evaluated in the ER.

Orthopaedic surgeons and sports medical doctors should consider the potential positive results for the application of this useful tool during the decision-making process. They should explain the risks and benefits of each therapeutic approach to their patients and choose the proper treatment for each one. For daily clinical practice, based on our results, we suggest not overusing surgery but to adopt an individualised treatment based on shared decision making guided by radiographic features and in accordance with patients’ needs.

Finally, well-designed, large-scale, randomised and prospective controlled trials including many patients are needed to better clarify this debated topic and to identify those patients who are more likely to develop non-union and would benefit from early surgery.

## Figures and Tables

**Table 1:** Socio-demographic, clinical and radiological characteristics at baseline and outcomes at last follow up of the overall population.

Variable	Patients Enrolled *n* = 134
**Socio-Demographic and Clinical Variables**	
Age, mean (SD), median (IQR)	44.20 (14.80), 46 (31–55)
Gender, number (%)	
Male	114 (85.1%)
Female	20 (14.9%)
BMI, mean (SD)	24.63 (2.55)
Smoking status, number (%)	
Active	60 (44.8%)
Inactive	74 (55.2%)
Type of trauma, number (%)	
Bike fall	48 (35.8%)
Motorcycle trauma	38 (28.4%)
Sports injury	27 (20.1%)
Simple fall	21 (15.7%)
Dominant side involved, number (%)	62 (46.3%)
**Radiological variables**	
Type of fracture, number (%)	
A1	11 (8.2%)
A2	25 (18.7%)
A3	17 (12.7%)
B1	6 (4.5%)
B2	29 (21.6%)
B3	44 (32.8%)
C1	2 (1.5%)
Initial shortening (%), mean (SD)	6.28 (5.14)
Residual shortening (%), mean (SD)	4.62 (4.60)
Initial displacement (%), mean (SD)	121.43 (42.48)
Residual displacement (%), mean (SD)	102.63 (35.85)
**Outcomes at last follow up**	
Constant score, mean (SD)	
Total	95.34 (6.24)
Pain subscale	14.28 (1.39)
Activities of Daily Living subscale	19.25 (1.66)
Range of movement subscale	39.09 (1.71)
Strength subscale	22.73 (2.94)
qDASH score, mean (SD)	
Total	4.87 (7.15)
Work	4.43 (9.41)
Sports	5.92 (11.25)
Return to work (months), mean (SD)	2.61 (0.97)
Return to sports (months), mean (SD)	4.45 (1.85)
VAS satisfaction, mean (SD)	7.59 (1.08)

SD = standard deviation; IQR = interquartile range; BMI = body mass index; qDASH = Quick Disabilities of the Arm, Shoulder and Hand; VAS = visual analogic scale.

**Table 2:** Socio-demographic, clinical and radiological characteristics and outcomes broken down according to the type of management (conservative versus surgical treatment).

Variable	Patients Treated Conservatively *n* = 75	Patients Undergoing Surgery *n* = 59	Statistical Significance
**Socio-Demographic and Clinical Variables**			
Age, mean (SD), median (IQR)	42.84 (13.70), 44 (30–54)	45.93 (16.04), 46 (33–58)	*p* = 0.2769
Gender, number (%)			*p* = 0.3796
Male	62 (54.4%)	52 (45.6%)	
Female	13 (65.0%)	7 (35.0%)	
BMI, mean (SD)	24.06 (2.27)	25.36 (2.72)	*p* = 0.0027
Smoking status, number (%)			*p* = 0.8392
Active	33 (55.0%)	27 (45.0%)	
Inactive	42 (56.8%)	32 (43.2%)	
Type of trauma, number (%)			*p* = 0.5773
Bike fall	29 (60.4%)	19 (39.6%)	
Motorcycle trauma	21 (55.3%)	17 (44.7%)	
Sport injury	16 (59.3%)	11 (40.7%)	
Simple fall	9 (42.9%)	12 (57.1%)	
Dominant side involved, number (%)	31 (50.0%)	31 (50.0%)	*p* = 0.1981
**Radiological variables**			
Type of fracture, number (%)			*p* = 0.0251
A1	5 (45.5%)	6 (54.5%)	
A2	20 (80.0%)	5 (20.0%)	
A3	6 (35.3%)	11 (64.7%)	
B1	3 (50.0%)	3 (50.0%)	
B2	13 (44.8%)	16 (55.2%)	
B3	28 (63.6%)	16 (36.4%)	
C1	0 (0.0%)	2 (100.0%)	
Initial shortening (%), mean (SD)	5.36 (4.61)	7.46 (5.56)	*p* = 0.0316
Residual shortening (%), mean (SD)	3.41 (3.60)	6.14 (5.27)	*p* = 0.0035
Initial displacement (%), mean (SD)	112.96 (43.43)	132.20 (39.00)	*p* = 0.0087
Residual displacement (%), mean (SD)	90.55 (28.01)	117.98 (38.95)	*p* < 0.0001
**Outcomes**			
Constant Score, mean (SD)			
Total	96.77 (5.56)	93.53 (6.62)	*p* < 0.0001
Pain subscale	14.57 (1.23)	13.90 (1.51)	*p* = 0.0003
Activities of Daily Living subscale	19.57 (1.25)	18.85 (2.01)	*p* = 0.0022
Range of movement subscale	39.33 (1.55)	38.78 (1.86)	*p* = 0.0210
Strength subscale	23.35 (3.10)	21.95 (2.54)	*p* < 0.0001
qDASH score, mean (SD)			
Total	4.24 (6.34)	5.66 (8.05)	*p* = 0.1466
Work	3.50 (9.09)	5.62 (9.76)	*p* = 0.0329
Sports	5.17 (11.82)	6.89 (10.49)	*p* = 0.0846
Return to work (months), mean (SD)	2.52 (1.15)	2.73 (0.67)	*p* = 0.0420
Return to sports (months), mean (SD)	4.08 (1.80)	4.93 (1.83)	*p* = 0.0011
VAS satisfaction, mean (SD)	7.64 (1.01)	7.53 (1.18)	*p* = 0.5798

SD = standard deviation; IQR = interquartile range; BMI = body mass index; qDASH = Quick Disabilities of the Arm, Shoulder and Hand; VAS = visual analogic scale.

**Table 3:** Major findings from the multivariate analysis of covariance (MANCOVA).

	Value	F	*p*
Type of treatment	0.2193	2.374	0.012
Type of fracture	0.7713	1.314	0.056
Initial shortening	0.1664	1.687	0.088
Initial displacement	0.1501	1.494	0.147

Pillai’s Trace is reported.

**Table 4:** Detailed impact of each independent variable on the outcomes from the multivariate analysis of covariance (MANCOVA).

	Dependent Variable	F	*p*
Type of treatment	Total Constant Score	20.42406	<0.001
	Pain	11.98866	<0.001
	ADL	9.14548	0.003
	ROM	5.74333	0.018
	Strength	13.38981	<0.001
	Total qDASH	1.79966	0.183
	Work qDASH	2.45299	0.120
	Sports qDASH	0.83182	0.364
	Return to work	1.68801	0.197
	Return to sports	7.84991	0.006
	VAS satisfaction	0.53103	0.468
Initial shortening	Total Constant Score	7.30993	0.008
	Pain	5.19306	0.025
	ADL	4.62164	0.034
	ROM	2.57767	0.111
	Strength	1.55391	0.215
	Total qDASH	11.86746	<0.001
	Work qDASH	6.93256	0.010
	Sports qDASH	4.22041	0.042
	Return to work	5.33146	0.023
	Return to sports	2.57716	0.111
	VAS satisfaction	6.49618	0.012
Initial displacement	Total Constant Score	4.23935	0.042
	Pain	0.12290	0.727
	ADL	0.80397	0.372
	ROM	1.81874	0.180
	Strength	6.01972	0.016
	Total qDASH	1.13655	0.289
	Work qDASH	0.93289	0.336
	Sports qDASH	0.08674	0.769
	Return to work	1.24169	0.268
	Return to sports	0.72554	0.396
	VAS satisfaction	0.04729	0.828

ADL = Activities of Daily Living; ROM = range of motion; qDASH = Quick Disabilities of the Arm, Shoulder and Hand; VAS = visual analogic scale.

**Table 5:** Effect sizes of the impact of the type of treatment (early surgery versus conservative management) on the outcome variables.

Variable	OR	95% CI
Pain	0.02	0.00 to 0.13
ADL	0.01	0.00 to 0.13
ROM	0.11	0.02 to 0.52
Strength	0.00	0.00 to 0.03
Total constant score	0.04	0.01 to 0.21
qDASH	5.47	1.48 to 20.18
qDASH work	15.05	3.44 to 65.93
qDASH sport	22.18	4.72 to 104.13
Return to work	2.90	0.99 to 8.47
Return to sports	9.35	2.18 to 40.03
VAS satisfaction	0.47	0.15 to 1.44

ADL = Activities of Daily Living; ROM = range of motion; qDASH = Quick Disabilities of the Arm, Shoulder and Hand; VAS = visual analogic scale.

## Data Availability

Data are available on request; contact the corresponding authors.

## Supplementary Materials

The following supporting information can be downloaded at: https://www.mdpi.com/article/10.3390/healthcare11131883/s1, Table S1: Socio-demographic, clinical, and radiological characteristics and outcomes of the study population broken down according to the type of trauma. Table S2: Major findings from the multivariate analysis of covariance (MANCOVA). Table S3: Detailed impact of each independent variable on the outcomes from the multivariate analysis of covariance (MANCOVA).
