# Supplementary material for: An Institutional Standardised Protocol for the Treatment of Acute Displaced Midshaft Clavicle Fractures (ADMCFs): Conservative or Surgical Management for Active Patients?
Source: Healthcare (Basel). 2023 Jun 29;11(13):1883. doi: 10.3390/healthcare11131883 (PMC10341159; doi:10.3390/healthcare11131883)
Supplement: Supplementary file 1 [file healthcare-11-01883-s001.zip › healthcare-2373944-supplementary.pdf]

Supplementary file

An institutional standardised protocol for the treatment of acute displaced midshaft clavicle fractures (ADMCFs): conservative or surgical management for active patients?

Carlo Biz [1,2], Assunta Pozzuoli [1,2,3*], Elisa Belluzzi [1,2,3*], Davide Scucchiari [1], Nicola Luigi Bragazzi [4], Alessandro Rossin [1], Mariachiara Cerchiaro [1], and Pietro Ruggieri [1,2]

Table S1. Socio-demographic, clinical, and radiological characteristics and outcomes of the study population broken down according to the type of trauma.

Variable	Patients without a sports trauma n=107	Patients with a sports trauma n=27	P – value
**Socio-demographic and clinical variables**			
Age, mean (SD), median (IQR)	47.53 (13.88), 48 (38-58)	31.00 (10.41), 29 (23-36)	p < 0.0001
Gender, number (%)			p = 0.2216
Male	89 (78.1%)	25 (21.9%)	
Female	18 (90.0%)	2 (10.0%)	
BMI, mean (SD)	24.80 (2.71)	23.96 (1.63)	p = 0.3316
Smoking status, number (%)			p = 0.0281
Active	53 (88.3%)	7 (11.7%)	
Inactive	54 (73.0%)	20 (27.0%)	
Dominant side involved, number (%)	47 (75.8%)	15 (24.2%)	p = 0.2806
**Radiological variables**			
Type of fracture, number (%)			p = 0.5362
A1	10 (90.9%)	1 (9.1%)	
A2	20 (80.0%)	5 (20.0%)	
A3	14 (82.4%)	3 (17.6%)	
B1	6 (100.0%)	0 (0.0%)	
B2	20 (69.0%)	9 (31.0%)	
B3	35 (79.5%)	9 (20.5%)	
C1	2 (100.0%)	0 (0.0%)	
Initial shortening (%), mean (SD)	6.07 (5.15)	7.15 (5.08)	p = 0.3241
Residual shortening (%), mean (SD)	4.48 (4.58)	5.17 (4.76)	p = 0.4666
Initial displacement (%), mean (SD)	118.86 (40.45)	131.63 (49.26)	p = 0.2738
Residual displacement (%), mean (SD)	102.32 (37.86)	103.85 (26.96)	p = 0.7510
**Treatment**			
Conservative	59 (78.7%)	16 (21.3%)	p = 0.7011
surgery	48 (81.4%)	11 (18.6%)	
**Outcomes**			
Constant score, mean (SD)			
Total	94.86 (6.54)	97.26 (4.49)	p = 0.0394
Pain subscale	14.23 (1.42)	14.44 (1.281)	p = 0.2323
Activity Daily Living subscale	19.12 (1.81)	19.78 (0.641)	p = 0.0887

Range of movement subscale	38.99 (1.83)	39.48 (1.051)	p = 0.3183
Strength subscale	22.52 (3.00)	23.56 (2.55)	p = 0.0613
qDASH score, mean (SD)			
Total	5.36 (7.68)	2.90 (4.043)	P = 0.2348
Work	4.79 (9.92)	3.01 (7.014)	p = 0.4402
Sports	5.84 (11.00)	6.25 (12.38)	p = 0.8590
Return to work (months), mean (SD)	2.61 (1.03)	2.59 (0.73)	p = 0.7916
Return to sports (months), mean (SD)	4.50 (1.96)	4.29 (1.34)	P = 0.9931
VAS satisfaction, mean (SD)	7.60 (1.10)	7.54 (1.05)	P = 0.9403

SD = Standard Deviation; IQR = Interquartile Range; BMI = Body Mass Index; qDASH = Quick Disabilities of the Arm, Shoulder and Hand; VAS = Visual Analogic Scale.

Table S2. Major findings from the multivariate analysis of covariance (MANCOVA).

		value	F	P
Type of treatment	Pillai's Trace	0.2193	2.374	0.012
	Wilks' Lambda	0.781	2.374	0.012
	Hotelling's Trace	0.2808	2.374	0.012
	Roy's Largest Root	0.2808	2.374	0.012
Type of fracture	Pillai's Trace	0.7713	1.314	0.056
	Wilks' Lambda	0.425	1.321	0.055
	Hotelling's Trace	0.9547	1.321	0.053
	Roy's Largest Root	0.3808	3.392	< .001
Initial displacement	Pillai's Trace	0.1501	1.494	0.147
	Wilks' Lambda	0.850	1.494	0.147
	Hotelling's Trace	0.1767	1.494	0.147
	Roy's Largest Root	0.1767	1.494	0.147
Initial shortening	Pillai's Trace	0.1664	1.687	0.088
	Wilks' Lambda	0.834	1.687	0.088
	Hotelling's Trace	0.1996	1.687	0.088
	Roy's Largest Root	0.1996	1.687	0.088

Table S3. Detailed impact of each independent variable on the outcomes from the multivariate analysis of covariance (MANCOVA).

	Dependent Variable	F	P
Type of treatment	Pain	11.98866	< .001
	ADL	9.14548	0.003
	ROM	5.74333	0.018

	Dependent Variable	F	P
	Strength	13.38981	< .001
	Total constant score	20.42406	< .001
	Total qDASH	1.79966	0.183
	Work qDASH	2.45299	0.120
	Sport qDASH	0.83182	0.364
	Return to work	1.68801	0.197
	Return to sport	7.84991	0.006
	VAS satisfaction	0.53103	0.468
Type of fracture	Pain	0.88592	0.508
	ADL	1.14063	0.344
	ROM	3.70879	0.002
	Strength	3.26407	0.006
	Total constant score	2.65762	0.019
	Total qDASH	0.94343	0.468
	Work qDASH	1.88745	0.090
	Sport qDASH	1.08938	0.374
	Return to work	1.30293	0.263
	Return to sport	1.73116	0.121
	VAS satisfaction	2.10288	0.059
Initial displacement	Pain	0.12290	0.727
	ADL	0.80397	0.372
	ROM	1.81874	0.180
	Strength	6.01972	0.016
	Total constant score	4.23935	0.042
	Total qDASH	1.13655	0.289
	Work qDASH	0.93289	0.336
	Sport qDASH	0.08674	0.769
	Return to work	1.24169	0.268
	Return to sport	0.72554	0.396
	VAS satisfaction	0.04729	0.828
Initial shortening	Pain	5.19306	0.025
	ADL	4.62164	0.034
	ROM	2.57767	0.111

Dependent Variable	F	P
Strength	1.55391	0.215
Total constant score	7.30993	0.008
Total qDASH	11.86746	< .001
Work qDASH	6.93256	0.010
Sport qDASH	4.22041	0.042
Return to work	5.33146	0.023
Return to sport	2.57716	0.111
VAS satisfaction	6.49618	0.012

ADL = Activity Daily Living; ROM = Range of Motion; qDASH = Quick Disabilities of the Arm, Shoulder and Hand; VAS = Visual Analogic Scale.
